# Behind the scenes of caregiving in patients with advanced cancer: A qualitative study on family caregivers

**DOI:** 10.1016/j.apjon.2023.100330

**Published:** 2023-10-30

**Authors:** Mahnaz Bahrami, Ahmad Nasiri

**Affiliations:** a Department of Medical-Surgical Nursing, School of Nursing and Midwifery, Student Research Committee, Birjand University of Medical Sciences, Birjand, Iran; b Department of Medical-Surgical Nursing, School of Nursing and Midwifery, Birjand University of Medical Sciences, Birjand, Iran

**Keywords:** Advanced cancer, Caregiving, Concerns, Qualitative study

## Abstract

**Objective:**

Family caregivers may have concerns that they do not want to disclose to others. This study aims to delve into the concealed facets of care provided by family caregivers to patients with advanced cancer.

**Methods:**

A qualitative approach was conducted in Iran from June 2022 to February 2023. Face-to-face, in-depth semi-structured interviews were carried out with 16 Iranian family caregivers of patients with advanced cancer, employing purposive sampling. The gathered data were analyzed using conventional content analysis, and Lincoln and Guba's criteria for rigor were applied to ensure the study's trustworthiness.

**Results:**

The analysis of the data resulted in the identification of three main themes with nine subthemes. The key themes derived from the experiences of family caregivers encompassed (1) chaotic mentalities, (2) troubling interdependence, and (3) desperational emotions.

**Conclusions:**

Caregivers of patients with advanced cancer commonly experience a highly fragile psychological state and are profoundly impacted by their concerns for the patient. Healthcare systems should prioritize ensuring that caregivers receive sufficient support and care.

## **Introduction**

Cancer is a pressing and rapidly growing global concern.^1^ Consequently, the burden of cancer incidence has been steadily increasing globally, including in Asian populations.^2^ By comparing national estimates of cancer incidence rates to those of other countries globally, Iran can be characterized as an intermediate-risk region for cancer, with an incidence rate of 152.7 per 100,000 individuals in 2020. According to GLOBOCAN estimates, assuming rates remain unchanged, the number of new cancer cases in Iran is projected to rise by 17.3% by 2025, reaching nearly 154,000 cases.^3^ Therefore, an increase in the number of families compelled to live with and care for cancer patients is expected.^4^ Currently, it is estimated that approximately 4.6 million individuals are responsible for providing care for their family members suffering from cancer within the confines of their own homes.^5^

Caregivers, as members of family or friends, provide unpaid support to individuals with chronic diseases such as cancer. They have various responsibilities from the time of diagnosis and throughout the cancer trajectory.^6^, ^7^, ^8^, ^9^, ^10^, ^11^ Family caregivers, who undertake this role unconditionally without objection or opposition, frequently disregard their own mental well-being and emotions to concentrate entirely on their loved ones who are approaching the end of life.^12^

Although informal caregivers are a major source of care for patients with advanced cancer, they often experience physical, emotional, social, and practical pressures.^13^, ^14^, ^15^ They frequently encounter an excessive workload due to the weighty responsibilities associated with managing patients' symptoms, providing emotional support, and aiding with caregiving tasks. Simultaneously, the burden on family caregivers is related to their experiences, which encompass physical, emotional, social, and economic challenges.^12^

A significant prevalence of emotional distress, ranging from 20% to 50%, has been reported among caregivers of cancer patients. Cancer and the therapeutic interventions implemented can introduce a multifaceted assortment of lifestyle modifications and emotional responses that can prove arduous for family members.^8^ Given that the role of caregivers during end-of-life care is frequently unprecedented, it can be perceived as intimidating by informal caregivers, potentially resulting in significant emotional distress.^16^

Limonero and Malhotra believe that both patients with advanced cancer and their caregivers experience numerous negative emotions, including but not limited to fear, worry, anxiety, anger, and grief, and neglecting to address these negative emotions may lead to suffering and a low quality of life.^17^^,^^18^ In this regard, a meta-synthesis of qualitative studies that focused on the experiences of informal caregivers of cancer patients (aged 19 to 85) described a high prevalence of negative emotions including the loss of joy, loneliness, hopelessness, and a sense of being trapped at home.^19^

Unfortunately, many caregivers who encounter advanced cancer are neither supported nor prepared.^7^ Family caregivers not only have responsibilities for managing patients' needs but also require support throughout the treatment trajectory or when confronted with their own concerns and emotions.^17^ Regrettably, most healthcare systems do not provide adequate support to caregivers.^20^ In fact, caregiving concerns increase fatigue among caregivers of patients with cancer.^21^ However, no studies centered on the emotional concerns of family caregivers of patients with advanced cancer were detected using qualitative research methods. Therefore, it is crucial to identify the most pressing concerns, needs, priorities, and preferences of caregivers.^22^ The concerns and anxieties of family caregivers of patients with advanced cancer are rooted in deep human emotions that cannot be discovered through quantitative research. Thus, a qualitative methodology is imperative to clarify their emotional concerns related to caregiving. Therefore, the present study aimed to explore the experiences of family caregivers of patients with advanced cancer regarding the hidden emotional concerns associated with caregiving for loved ones.

## Methods

### Study design

This study was conducted using a qualitative approach, specifically content analysis.^23^ This approach provides the best opportunity for a better understanding of phenomena and the exploration of life experiences in the realm of social and cultural events.^24^ Furthermore, it facilitates the development of empirical, conceptual, and theoretical frameworks for both research and practical applications.^25^

#### Setting and participants

Participants in this study were selected through purposive sampling of caregivers of cancer patients attending an oncology care center in Birjand. The inclusion criteria were being a caregiver for an advanced stage 3 or 4 or metastatic cancer patient for at least 6 months, being at least 18 years old, living with the patient or providing a minimum of 3 h of daily care to the patient, and willingness to participate in the study. Any person who expressed an unwillingness to continue collaborating was excluded from the study. The researcher endeavored to identify and select participants who possessed valuable information or experience related to the phenomenon under investigation.^26^

### Data collection

Data were gathered between June 2022 and February 2023 through in-depth, semi-structured interviews that were conducted in a face-to-face format. This method is widely regarded as the most effective for qualitative studies.^24^ The interviews occurred in a quiet and private setting and began with a general question: “Please talk about your concerns and worries as a caregiver for advanced cancer patients.” To further clarify aspects related to the main topic, exploratory questions were utilized such as “Please elaborate further?” and “What do you mean by that?” The interviews lasted between 30 and 90 minutes, and 16 individual interviews were conducted with family caregivers until data saturation were achieved. In qualitative research, data saturation are reached when no new categories emerge and previously collected information is repeated and confirmed.^24^^,^^27^ Field notes were also taken after each interview, and all the recorded interviews were repeatedly listened to by the primary researcher and transcribed verbatim in Persian.

### Data analysis

To analyze the research data, a qualitative content analysis approach was utilized through the methodology proposed by Graneheim and Lundman.^27^ The text of each interview was examined repeatedly to gain a better understanding of the participants' experiences. Within the transcripts, meaningful units containing important or relevant information were condensed to generate initial codes. Following this, the initial codes were meticulously analyzed and grouped into subcategories based on their conceptual similarities. Through an inductive approach, these subcategories were further classified into main categories. The coding process and the emergence of the main themes were reviewed and discussed by the authors undergoing the study. Subsequently, the categories were established as representations of the contractual content of the transcripts. Ultimately, three themes and nine sub-themes were identified. The entire coding and data analysis process was conducted using the MAXQDA22 software.

### Ethical considerations

This study adhered to the principles outlined in the Declaration of Helsinki. Approval was obtained from the Ethics Committee of Birjand University of Medical Sciences (IRB No. Jun.20.2022/IR.BUMS.REC.1401.140). Written informed consent was secured from all participants prior to their participation in the study, with audio recordings also being utilized.

### Trustworthiness

To ensure the trustworthiness of the data, the four criteria of credibility, confirmability, dependability, and transferability proposed by Guba and Lincoln were employed.^24^ The credibility of the study was validated by establishing effective interactions with the participants through in-depth interviews and allocating sufficient time (nine months) for data collection. The dependability of the study was ensured by documenting and recording all stages of the research, from data collection to data analysis. Additionally, the use of personal notes as a technique helped to maintain the stability and continuity of the findings. The confirmability of the data were verified by providing coded interviews to the participants for conformity with their experiences and by having a proficient qualitative researcher review the codes and extract concepts. To enhance transferability, purposive sampling was conducted to maximize the diversity of participants in terms of gender, age, kinship relationship with the patient, occupation, caregiver education, and type of cancer. Furthermore, we endeavored to provide detailed explanations of the study methodology for future research.

## Results

Sixteen Iranian family caregivers of patients with advanced cancer participated in the present study. The participants' demographic characteristics are summarized in Table 1.

**Table 1:** Characteristics of family caregiver of advanced cancer patients.

No	Caregiver's gender	Caregiver's age (years)	Relative to the patient	Duration of care	Caregiver's job	Caregiver's education	Patient's gender	Patient's age (years)	Type of cancer
P1	Female	39	Sister	2 years	Housekeeper	Bachelor's degree	Female	32	Cervix
P2	Female	46	Wife	7 months	Worker	Diploma	Male	46	Osteosarcoma
P3	Male	26	Son	2 years	Military	Diploma	Female	54	Esophageal
P4	Female	33	Wife	1 year	Housekeeper	Diploma	Male	34	Colon
P5	Male	35	Husband	7 months	Worker	Diploma	Female	32	Brain
P6	Male	41	Husband	6 months	Employed	Bachelor's degree	Female	42	Brest
P7	Female	39	Sister	2 year	Housekeeper	Bachelor's degree	Female	32	Cervix
P8	Female	34	Daughter	1 year	Housekeeper	Diploma	Male	64	Esophageal
P9	Female	43	Stepmother	1 year	Housekeeper	Uneducated	Female	18	Leukemia
P10	Male	55	Husband	1 year	Retired	Diploma	Female	54	Brest
P11	Female	31	Mother	1.5 years	Employed	Associate degree	Female	3.5	Cerebellum
P12	Female	46	Mother	2 years	Housekeeper	Bachelor's degree	Female	19	Osteosarcoma
P13	Male	37	Husband	1 year	Military	Diploma	Female	38	Brest
P14	Female	46	Wife	2 years	Teacher	Bachelor's degree	Male	67	Chordoma
P15	Female	52	Wife	6 months	Housekeeper	Uneducated	Male	55	Leukemia
P16	Female	34	Daughter	8 months	Teacher	Bachelor's degree	Female	56	Colon

Following the content analysis of the interviews, three main categories were extracted: (1) chaotic mentalities; (2) troubling interdependence; and (3) desperational emotions. A comprehensive exposition of each main category and sub-category is presented in Table 2. The main categories are explained as follows.

**Table 2:** Themes and Sub themes.

Theme	Subtheme
Chaotic mentalities	Annoying by recalling memories
	Pity for the patient's loved ones
	Apprehension of possible future challenges
	Worrying about getting cancer
Troubling interdependence	Deep emotional attachment to the patient
	Exclusive trust in care by caregiver
	Self-neglect while extreme commitment to patient
Desperating emotions	A sense of inefficiency
	Mental weariness

### Chaotic mentalities

One of the most significant concerns for caregivers of patients with cancer is their chaotic mentality and excessive mental worries. Several factors contribute to caregivers’ mental turmoil. Undoubtedly, this abundance of thoughts and worries significantly confuses the minds of family caregivers of patients with advanced cancer.

#### Annoying by recalling memories

The act of reminiscing about the patient's memories prior to cancer and contrasting them with their present state can be particularly annoying for caregivers, especially cancer patients who have unique physiological and psychological conditions due to treatment complications.

*“Now my wife is so sick. When she used to live like everyone else, talking and joking, our life was good. I remember it, and it feels like my brain wants to collapse. This annoys me" P10*

*“My daughter is not the same anymore. She used to be so sweet and attractive that whenever she talked to others, they would fall for her because she was very warm and friendly, but now… It bothers me to bring up these memories.” P12*

*“I say to myself, I wish my wife did not get cancer. Ever since I saw my wife's picture on her brother's phone, I get so annoyed that I get a headache when I remember it, and I say, ‘Oh my God, can I see my wife walking on her own two feet in our own house one more time?” P13*

#### Pity for the patient's loved ones

Additionally, caregivers are responsible not only for the patient's well-being but also have concerns for other family members. They are not only apprehensive about the patient's condition but also about the implications of the patient's cancer for other family members.

*“One of the greatest concerns and worries that weigh heavily on my mind is how to explain my sister's critical condition to my parents. They would be devastated upon hearing this news, especially my father, who loves my sister very much.” P7*

*“Regarding my children, I have serious concerns. I take them to their grandparents' homes in the village to divert their focus from their mother's illness. They are more at ease there.” P13*

*“It's really tough. My sister's illness is proving to be a great burden for my father, who is struggling with heart disease. I always think about how I would tell my father if something happened to my sister. This situation has truly created a challenge for me.” P1*

#### Apprehension of possible future challenges

Furthermore, the majority of caregivers anticipated the death of their loved one, causing great disruption to their thoughts regarding what the future may hold. They regularly engage in imagining a future that has not yet come to pass and scenarios that they have not yet encountered.

*“I often think about what would happen if I were to lose him one day… Would I be sufficient for this life if my husband were to pass away? Can I raise our children better than this?” P4*

*“My nephews are very mischievous. Occasionally, my sister has trouble handling them, and I am persistently concerned regarding how their upbringing and future will be if something were to happen to my sister.” P1*

*“I'm continuously worried about what will happen to my life and my kids; therefore, I'm constantly worrying about the future. It’s really hard since I can't come to any firm conclusions or answers.” P15*

*“In addition to myself, my spouse is also constantly worried about the future, wondering what I would do if he passed away. How would I provide for our lives? On the other hand, my children would have been deeply affected without their father. My daughters are very attached and dependent on their father.” P2*

#### Worrying about getting cancer

Another significant emotional concern that profoundly affects caregivers is the fear of their own or their loved ones' cancer diagnoses. The fear of developing cancer prompts them to interpret even the slightest symptoms that they or others exhibit as signs of cancer, leading to stress and anxiety.

*“I constantly think that I might also get cancer someday, or maybe I already have it. I always fear checkups for myself because I fear they might tell me that I have cancer too.” P8*

*“Sometimes, my mind becomes preoccupied with the fear of cancer. I find myself constantly contemplating the possibility that my children will inherit this disease, especially since medical professionals have mentioned its hereditary nature. I pray to God that my children never have to deal with this disease.” P4*

*“During the days when my spouse was undergoing radiation therapy, as I awaited her, I thought that perhaps I had contracted cancer. It seemed as though an inner voice was whispering to me that I too was afflicted with cancer.” P6*

*“I’m habitually tormented by worry, and each time I encounter a sign or symptom, I get anxious about the plausibility of having cancer. For example, amid my shower, I touch my breasts and tell myself that I have breast cancer or that I am without a doubt suffering from uterine cancer.” P16*

### Troubling interdependence

There is a strong relationship between the caregiver and the patient during caregiving, which can be intense and troublesome on numerous occasions.

#### Deep emotional attachment to the patient

The caregiver exhibits deep concern for the patient's well-being to such an extent that separation from the patient becomes inconceivable, and the caregiver must be present at the patient's bedside. Even when urged to take a break, their minds remain involved with the patient's condition, and they have an inclination to return to the patient's bedside as soon as possible.

*“I can't stay away from her. After 7–8 hours of not seeing her, I feel the need to go and see her. This caused me great distress to be separated from her. Even if there is nothing to be done in her presence, I find it unbearable to be apart from her.” P7*

*“My brother-in-law would come here and say, "I'll be with the patient tonight." I would tell him that I prefer to stay here instead of going home and thinking about what might happen to the patient. I cannot bear to be separated from her, even for a moment. When I'm beside her, I feel calmness.” P10*

*“I don't allow anyone else to take care of her. Wherever I go, my heart remains here. I want to return her as quickly as possible. I can't bear to leave her alone for even a moment.” P3*

*“I'm with her throughout the entire 8–9 days she is hospitalized, from the moment she arrives at the hospital until the moment she is discharged. Even though I get very tired during the entire hospital stay, I love her so much that I do not want anyone else to be with her. I want to be here myself.” P5*

#### Exclusive trust in care by caregiver

Conversely, patients were equally dependent on caregivers for their care. Thus, the patient is confident only in the care provided by the caregiver, and only the opinion of the caregiver is important to him/her in all cases related to his or her care. It seems that the patient trusts only the caregiver for their well-being and care.

*“I knew that she really trusted me when it came to anything related to her illness, and she felt better in my presence. She wants others to ask me whether something is good for her. To my surprise, she had more confidence in my words than medical professionals.” P7*

*“He consistently expresses that he experiences a sense of ease only when I take care of him. The moment I exit the room, he asks me to return. He doesn’t even let me go to the bathroom. He has become very dependent on me for care.” P6*

*“One day, when my cousin was in the hospital, my sister pleaded with her to have me come and be with her. My sister would say that I am a tower of strength for her. She had a lot of trust in me and was highly dependent on me.” P7*

*“She wants me to be by her side because she can rely on me and share everything with me. That is why, when I leave and someone else is with her, she complains and asks why I was not there. She believes that the other person who was present couldn't take care properly, and she trusts that I can provide the care she needs.” P1*

#### Self-neglect while extreme commitment to patient

However, this mutual dependency often leads to self-neglect among caregivers. The caregivers become so engrossed in the patients’ care that they forget about their role, personal lives, and even themselves, and it seems they have devoted themselves to the patient. This dependency, rather than a physical burden, places a significant psychological burden on caregivers. It mentally occupies their minds, causing emotional distress as they continually worry about the patient.

*“Even during my sleep, I do not have peace. My sleep is stressful. Every ten minutes, I wake up and wonder if my husband is breathing. Or isn't he in pain? I can't go anywhere. Both he and I have become sensitive, and I keep thinking he's sick.” P4*

*“Since taking on the role of caregiver, I have gradually become disconnected from the ebb and flow of daily existence. It is like I have forgotten myself. I forget what sleep and food are. What is life? I have distanced myself from all of these things.” P1*

*“I was here for 10–12 hours' yesterday, and my patient is in such a condition that you can't sit down. She constantly asks me to do things like lift her hand, help her walk, or massage her feet. She is very restless, and there are times when I feel like I am about to collapse. In this situation, you forget yourself. I have even forgotten about my own family.” P12*

### Desperation emotions

The nature of cancer, particularly in its advanced stages, engenders a sense of helplessness among caregivers, who may feel incapable of providing meaningful services or treatment to their patients.

#### A sense of inefficiency

As patients approach the end of life, caregivers may feel unable to improve their condition, exacerbating their sense of helplessness. This emotion deeply concerns caregivers, to the extent that they may experience restlessness.

*“It's very difficult for me; I see him before my eyes; he's getting weaker day by day and getting weaker, and I feel powerless since there's nothing I can do to progress his condition.” P13*

*“I feel as though there is nothing I can do to help my husband… I get home and just stare at the door and walls, tears flowing down my cheeks because I can't help him.” P2*

*“The worst thing that could have occurred to me was to feel stuck with no way out. You cannot do anything, nor can you stay at home. It feels like you're not calm or settled at all.” P1*

*“I see her deteriorating day by day, unable to eat anything. Her disease is advancing consistently, like a candle melting in front of us, but I can't help her.” P3*

#### Mental weariness

Additionally, owing to a sense of depletion, caregivers may encounter a loss of physical and emotional energy, which can negatively impact the quality of their caregiving.

*“This is especially noticeable among the offspring or fathers who care for their patients. When people reach the terminal stages of a disease, their ability to care for others tends to diminish. They experience psychological exhaustion, and they have every right to feel that way.” P9*

*“There were times when I couldn't bear it anymore during her treatment process. For example, when I received the pathology test results, I thought I was going mad. I feel empty inside. I sat in the backyard and cried loudly.” P10*

*“It's really difficult. No matter how hard I try, words cannot properly explain the great psychological and emotional burden I have been under during this period, especially since I did not want my family to know what was going on. Carrying the weight of everything on my shoulders became too much for me, and I couldn't take it any longer.” P11*

*“I'm totally smashed mentally, but no one gets it.” P14*

## Discussion

This study aimed to highlight the hidden emotional concerns of family caregivers associated with caring for their loved ones. Three major themes emerged from their experiences: chaotic mentalities, troubling interdependence, and desperational emotions.

### Participants in this study noted that they had turmoil at the end stage of cancer

In a review of past studies, the results of the Hashemi–Ghasemabadi study reported “being trapped with negative thoughts” as one of the experiences mentioned by caregivers who recently assumed the caregiver role.^9^ Therefore, caregivers experience turmoil thoughts from the time of diagnosis to the end stages of the disease, even though the nature and content of these concerns may change throughout the disease process.

Family caregivers of patients with advanced cancer in the present study were annoyed by recalling memories and observing the deteriorating condition of their patients. Similarly, Alam et al. considered the patient's deteriorating condition as a risk factor for adverse mental health outcomes in caregivers.^28^ According to Solberg et al., it was also difficult for caregivers to see the patient's transformation from an independent individual to a completely dependent and helpless person; this affected them emotionally, and they did not know where to turn with their thoughts.^29^

In the current study, caregivers experienced apprehension regarding possible future challenges. Studies reported that the family system faces a significant crisis when a loved one is in the end stage of cancer and approaches death, and it is an emotionally exhausting process. Among all human experiences, the death of a loved one or its impending arrival is the most difficult challenge for families and members within them.^30^^,^^31^ Martín et al. also suggested that as the patient's death approaches, family caregivers are concerned about how they will live without their spouse, wife, or children and how their lives will be; additionally, they are constantly worried about their own futures or the future of the affected family member.^32^ Findings from several studies have demonstrated that caregivers frequently worry about the patient's status and anticipate hearing the worst news regarding their patients at any time.^33^, ^34^, ^35^, ^36^ Consequently, it is important to continue supporting them even after the patient's death.^37^

Another chaotic mentality experienced by participants in this study was worrying about getting cancer. Jensen et al. state that the public perception of cancer is such that compared to other diseases that may be equally dangerous or serious, its diagnosis causes a person to feel more anxiety and fear.^21^ Solberg et al. reported that the word “cancer” evoked various emotions and reactions in family caregivers, with fear being the initial emotion that they experienced; on the other hand, they felt helpless and concerned about their health because of a lack of information and guidance from healthcare professionals, as the care was primarily focused on the patients rather than on them.^29^ Even oncology nurses mentioned that working in the oncology unit, due to dealing with the deaths of cancer patients, caused them to have negative thoughts about their health.^38^

### In the present study, we found that a bond between the caregiver and the patient is formed during the caregiving process, which goes beyond a simple family relationship

This mutual relationship frequently presents challenges to caregivers. Martin et al. believe that in many cases, the presence of an incurable disease impacts the caregiver–patient relationship and redefines it.^32^ Several studies have supported the development of a deep and mutual relationship between the caregiver and the patient throughout the caregiving process to the extent that the caregiver and the patient influence each other's mental and physical health^39^, ^40^, ^41^ and feel a sense of commitment to endure their current situation.^33^ Our study showed a mutual relationship between caregivers and patients, as evidenced by their insistence on physical presence. According to the study by Bei Physical proximity and attachment to another person are intimately intertwined, so that physical closeness to someone leads to them being perceived as a secure haven, improving feelings of safety and reducing distress and anxiety in stressful situations.^6^ In advanced cancer, caregivers serve as the primary source of physical, emotional, and social support for their loved ones.^35^^,^^41^ In contrast to the results of the present study, caregivers might sometimes be unwilling to provide care. Bridge et al. suggest that caregiver and care recipient factors can affect one's tendency to provide care; it appears that the strongest sense of commitment may exist among spouses or child caregivers.^42^

The majority of the participants in the present study forgot about themselves by being immersed in patient care, which was found to be a troubling interdependence experience. Hashemi-Ghasemabadi et al. highlighted that a complete commitment to providing care and support for the patient engulfs caregivers, causing them to neglect their own issues and concerns.^9^ Cultural norms, religious teachings, and strong family bonds in Iranian families imply an inclination towards providing high levels of care.^43^ Excessive attention to the patient comes at the cost of neglecting other roles and responsibilities, which leads to tension within the family. This could potentially result in the overall collapse of the family unit, as mentioned in the study by Nemati et al.^34^ In Martins' study, caregivers experienced a limited life. In fact, they often state that they may put aside their interests and professions, restrict or even completely abandon their social lives, and consider leisure time as something belonging to the past.^32^ Hejazi et al. mentioned that for hemodialysis patients, the hemodialysis schedule had an impact on all aspects of the caregiver's life, and caregivers were deprived of social activities and spent time with their families and friends.^33^

### Feeling helpless and subsequent inefficacy have been major concerns among caregivers, particularly given that the participants in the current study were caregivers of patients with advanced cancer

This feeling of powerlessness and inefficiency was profound. The participants in Hashemi-Ghasemabadi's study also perceived their current situation as critical and often felt unable to cope with challenges. They believed they were insufficient to solve issues and achieve caregiving goals.^9^ Also based on Astrup et al. and Kizza et al. studies, family caregivers experienced feelings of fear, uncertainty, hopelessness, fatigue, and even thoughts of death.^39^^,^^44^ According to the results of Nemati's study, the perception of insufficient provision of quality care depends on the knowledge, experience, and personal skills of the caregiver, as each caregiver has a specific capacity that cannot be exceeded.^34^ However, as patients reach the end stages of life, caregivers begin to realize that the end-of-life situation cannot be changed. They are aware that there will be an end, and there is nothing they can do to prevent it.^32^ Park et al. reported that more than one-fourth of caregivers felt some degree of uncertainty about what they should do for the patient.^45^

In Nemati's study, caregivers described themselves as immersed in a sea of grief and sorrow, where no safe haven is seen; they described the caregiving experience as being trapped and victimized, leading to a sense of helplessness.^34^ Studies conducted on caregivers of hemodialysis patients have also revealed feelings of despair, hopelessness, and exhaustion. They expressed being witnesses to the patient's suffering without being able to help them. In addition, the ongoing and lifelong nature of caregiving contributes to psychological fatigue in caregivers.^33^^,^^35^ De Padova et al. show that the emotional burden on caregivers is frequently higher than that on patients.^8^ As a result of increased caregiver burden, caregivers experience fatigue, sleep deprivation, insufficient time for personal care, lack of attention, distress, depression, crying, reluctance and unwillingness to talk, fear, hopelessness, decreased social interactions, and disruptions to family life.^46^ Family caregivers, similar to nurses, have the responsibility of caregiving. In the study by Moghadam, emotions of mental and physical exhaustion, chronic and ongoing stress, excessive fear of potential harm, and emotional depletion are all factors that contribute to caregivers being emotionally exhausted.^38^

### Limitations

The findings of this study should be interpreted in light of these limitations. As with all qualitative studies, the transferability of the results to other populations and settings is constrained. Specifically, our study was confined to examining emotional concerns in family caregivers of end-of-life cancer patients, thus making generalizations of the results across all treatment phases inappropriate.

## Conclusions

The results of the present study showed that caregivers of patients with advanced cancer are confronted with mental distress; they have a strong emotional relationship with the patient, which may result in trouble and ultimately lead to desperation. These caregivers appear to be engulfed by a sea of worries and concerns, but few express their emotional worries candidly and voluntarily. Caregivers and their concerns are usually not seen in health systems, and these unaddressed concerns have adverse consequences on the mental and physical health of patients and caregivers.^47^ Consequently, addressing caregivers’ psychosocial requirements can improve patient outcomes. The availability of social support networks can be instrumental in providing emotional assistance to caregivers of patients with cancer. This social support can help them cope with their situation and share their experiences with people who are not emotionally involved in the experience of this disease.

## Data Availability

The data that support the findings of this study are available from the corresponding author A.N upon reasonable request.

## CRediT author statement

**Mahnaz Bahrami:** Conceptualization; Data curation; Investigation; Methodology; Writing – original draft; **Ahmad Nasiri:** Conceptualization; Methodology; Project administration; Supervision; Writing – review & editing. All authors had full access to all the data in the study, and the corresponding author had final responsibility for the decision to submit for publication. The corresponding author attests that all listed authors meet authorship criteria and that no other meeting the criteria has been omitted.
